# Bioinformatic workflow fragment discovery leveraging the social-aware knowledge graph

**DOI:** 10.3389/fgene.2022.941996

**Published:** 2022-08-26

**Authors:** Jin Diao, Zhangbing Zhou, Xiao Xue, Deng Zhao, Shengpeng Chen

**Affiliations:** ^1^ School of Information Engineering, China University of Geosciences (Beijing), Beijing, China; ^2^ Computer Science Department, TELECOM SudParis, Evry, France; ^3^ School of Computer Software, College of Intelligence and Computing, Tianjin University, Tianjin, China; ^4^ Wuda Geoinformatics Co., Ltd., Wuhan, China

**Keywords:** bioinformatic workflow, fragment discovery, social relations, knowledge graph, scientific workflow

## Abstract

Constructing a novel bioinformatic workflow by reusing and repurposing fragments crossing workflows is regarded as an error-avoiding and effort-saving strategy. Traditional techniques have been proposed to discover scientific workflow fragments leveraging their profiles and historical usages of their activities (or services). However, social relations of workflows, including relations between services and their developers have not been explored extensively. In fact, current techniques describe invoking relations between services, mostly, and they can hardly reveal implicit relations between services. To address this challenge, we propose a social-aware scientific workflow knowledge graph (*S* ^2^ *KG*) to capture common types of entities and various types of relations by analyzing relevant information about bioinformatic workflows and their developers recorded in repositories. Using attributes of entities such as credit and creation time, the union impact of several positive and negative links in *S* ^2^ *KG* is identified, to evaluate the feasibility of workflow fragment construction. To facilitate the discovery of single services, a service invoking network is extracted form *S* ^2^ *KG*, and service communities are constructed accordingly. A bioinformatic workflow fragment discovery mechanism based on Yen’s method is developed to discover appropriate fragments with respect to certain user’s requirements. Extensive experiments are conducted, where bioinformatic workflows publicly accessible at the myExperiment repository are adopted. Evaluation results show that our technique performs better than the state-of-the-art techniques in terms of the precision, recall, and *F1*.

## 1 Introduction

**FIGURE 1:**
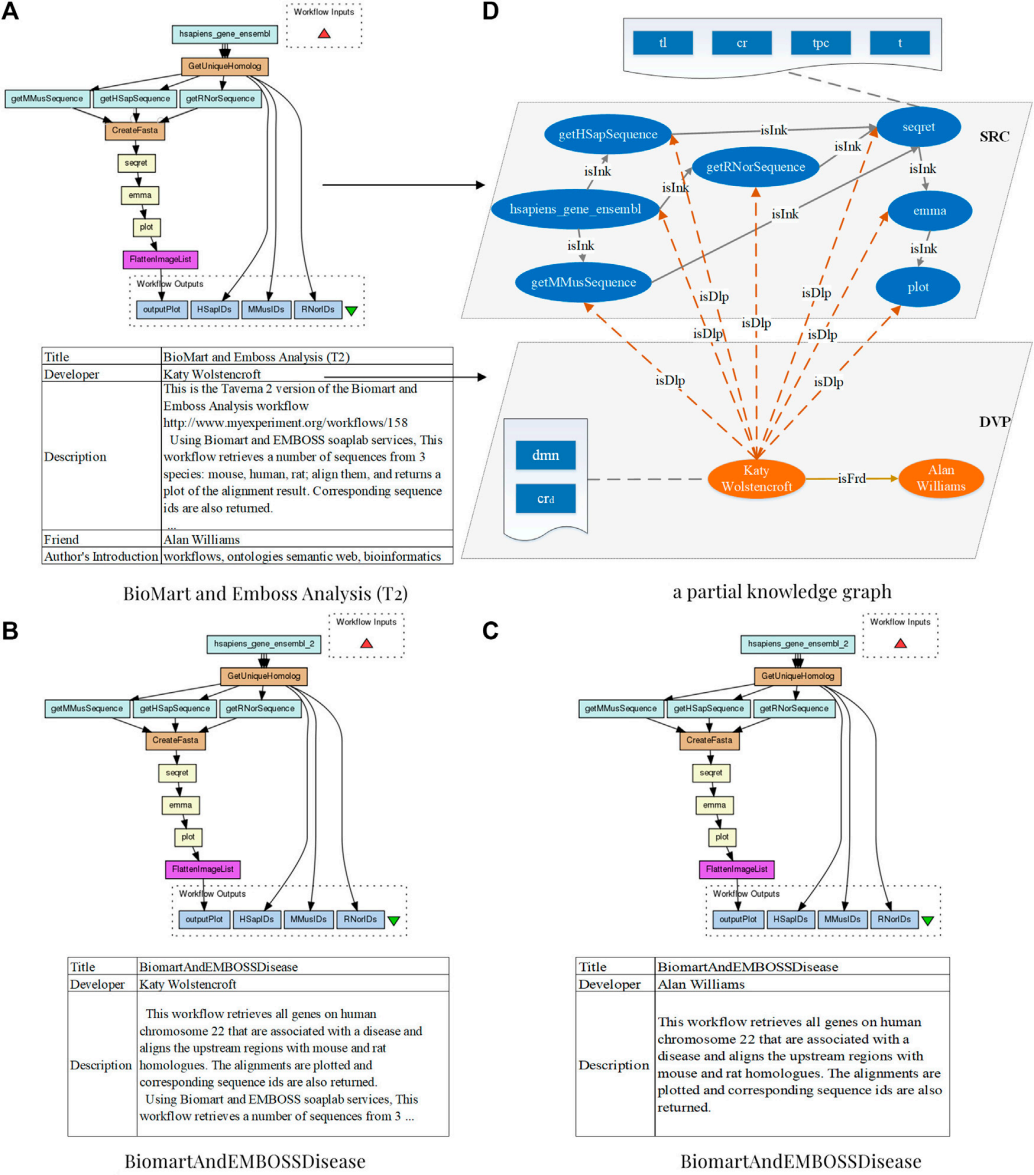
Three bioinformatic workflows from *Taverna* 2 of the myExperiment repository with the title “*BioMart and Emboss Analysis (T2)*”, “*BiomartAndEMBOSSDisease*”, and “*BiomartAndEMBOSSDisease*”, respectively, and a partial knowledge graph of the *BioMart and Emboss Analysis (T2)* in *S* ^2^ *KG*.

With the wide-adoption of web service technology, recurring data and computational resources are increasingly encapsulated as web services or mashup APIs and assembled as scientific workflows (Fischer et al., 2021; Coleman et al., 2022). Online repositories, such as *myExperiment* ^1^, are publicly accessible for publishing and sharing of scientific workflows constructed by scientists from various disciplines (Gkortzis et al., 2021). Bioinformatics, for example, has seen a spectacular rise in the availability of distributed services (Brandt et al., 2021) and allows rapid and accurate analysis using bioinformatic workflows. Examples of bioinformatic workflows from myExperiment are illustrated in Figure 1. With an increasing number of bioinformatic workflows available online, scientists can reuse and repurpose legacy workflows, rather than developing from scratch, to satisfy novel requirements which are examined to be completely or partially satisfiable by legacy workflows in repositories (Brandt et al., 2021; Rosa et al., 2021). As shown in Figure 1B, the workflow “BiomartAndEMBOSSDisease” retrieves all genes on human chromosome 22, which are associated with a disease, and aligns upstream regions with mouse and rat homologues. This workflow can be reused to reduce the cost when a scientist is willing to design a similar experiment. In fact, considering knowledge-intensiveness and error-proneness for constructing a novel bioinformatic workflow, reusing or repurposing current workflows has been evidenced as an error-avoiding and effort-saving strategy for conducting reproducible bioinformatics experiments (Ren and Wang, 2018; Almarimi et al., 2019). To facilitate the reuse and repurposing of bioinformatic workflows, techniques for discovering and recommending the most relevant fragments of current workflows are fundamental (Yao et al., 2021).

Current techniques have been developed to support the discovery of workflow fragments with similarity assessment. Traditionally, these works evaluate structural similarities between workflows (Bai et al., 2017; Zhang et al., 2018; Zhou et al., 2018), where partial-ordering relations specified upon services are concerned. Although the structure can well-represent the execution semantics of individual workflow fragments, semantic mismatches exist, due to domain differences of workflow developers. To mitigate this problem, annotation-based similarity computation techniques are proposed to complement the structural similarity assessment. Annotations are typically provided by developers to prescribe the category and essential functionalities of certain workflows (Ni et al., 2015; Zhong et al., 2016; Hao et al., 2017). Since workflows may not be accompanied with annotations in certain scenarios (Starlinger et al., 2014), annotation-based strategies with inaccurate similarity calculations may not work as expected. As a result, it may hardly recommend suitable fragments when performing certain scientific experiments.

Considering the fact that developers themselves, who prescribe the annotations, may provide insights about the execution relations between workflows, this study proposes to explore social relations between developers to facilitate recommending appropriate workflow fragments. Figure 1 shows a motivating example of two similar bioinformatic workflows, which are built by two developers who are actually friends. Therefore, incorporating the social relations of developers is promising to further improve the recommendation performance. Discovering fragments from bioinformatic workflows that are assembled by developers in social relations is a promising research challenge. While workflow repositories, such as myExperiment, have been constructed for decades, there still have insufficient socially relevant data on developers. As a result, current techniques focus on gathering and applying certain social information, such as developer reputation, to facilitate the discovery accuracy of appropriate workflows and services (Qiao et al., 2019; Khelloufi et al., 2021; Zhu et al., 2021). In fact, more relations between services (Herbold et al., 2021), and their positive or negative links on workflow fragments discovery and recommendation, have not been explored extensively. Therefore, considering social relevance between developers and services, for facilitating the reuse and repurposing of current workflow fragments, is a challenge to be explored further.

To address this challenge, this study proposes a novel workflow fragment discovery mechanism, by exploring social relations of developers and services that are formed in a knowledge graph. Major contributions presented in this article are summarized as follows:

• We constructed a social-aware scientific workflow knowledge graph (*S* ^2^ *KG*) from the myExperiment repository, where services and developers of bioinformatic workflows are encapsulated as entities, and relevant attributes of entities, such as topic, reputation, and domain, are obtained. In addition, multiple relations between entities, including (i) invocation relations between services, (ii) developer relations between services and their developers, and (iii) friend relations between developers, are captured.
• We proposed a novel bioinformatic workflow fragment discovery mechanism leveraging *S* ^2^ *KG.* Specifically, positive or negative links between services are identified by analyzing their credits, co-invocation possibilities, and co-developer relations (Ni et al., 2015). A service invoking network (SINet) is formed based on invocation relations between services in *S* ^2^ *KG.* Service communities are generated from SINet using the fast unfolding method (Blondel et al., 2008), to facilitate individual candidate services discovery from a functional perspective. Thereafter, services are pairwisely connected through query operations upon *S* ^2^ *KG.* The Yen’s method (Yen, 1971) is adopted to construct and recommend appropriate workflow fragments to satisfy user’s requirements.

Bioinformatic workflows in myExperiment are adopted as the data set in our experiments, where social relations between services and developers are discovered. Extensive experiments are conducted, and evaluation results show that our technique, which complements social relations, outperforms the state-of-the-art counterparts in terms of the precision, recall, and *F1*.

This study is organized as follows. Section 2 introduces relevant concepts of *S* ^2^ *KG* and the attributes of entities. Section 3 presents the process of workflow recommendation based on *S* ^2^ *KG*. Section 4 evaluates our method and makes a comparison with state-of-the-art techniques. Section 5 discusses related works. Section 6 concludes this study.

## 2 *S* ^2^ *KG* construction

This section introduces relevant concepts and presents the construction procedure of *S* ^2^ *KG*.

### 2.1 Concepts of *S* ^2^KG

*myExperiment* is an online research environment that supports social sharing of developers’ workflows (Goble et al., 2010), which consists of several services. According to these characteristics, the knowledge graph constructed on this repository in this study includes two types of entities, that is, services and developers, as well as three types of relations between them. The workflow is used to reflect the invocation relation between services, so it is not used as a separate entity. The specific definitions are as follows.

A service in *S* ^2^ *KG* is defined as follows:

Definition 1 *(Service). A service is a tuple src = (tl, tpc, cr, t), where:*

*• tl is the title of src;*
*• tpc is the topic vector that represents its functions;*
*• cr is its credit, which is calculated based on workflows containing this src;*
*• t represents the created time of src.*

A developer in *S* ^2^ *KG* is defined as follows:

Definition 2 *(Developer). A developer is a tuple dvp = (dmn, cr_d_), where:*

*• dmn is the topic vector representing his research domains;*
*• cr_d_ is the reputation calculated by his rating and the credit of his workflows.*

A social-aware scientific workflow knowledge graph (*S* ^2^ *KG*) is defined as follows:

Definition 3 *(*S* ^2^ *KG*). *S* ^2^KG is a tuple (V, LNK), where:*

*• V = SRC ∪ DVP is a set of entities for services, SRC, and a set of developers, DVP;*
*• LNK is a set of directed links which specify three kinds of relations: (i) services and services (isInk), (ii) services and developers (isDvp), and (iii) developers and developers (isFrd).*

A scientific workflow in S^2^KG is defined as follows:

Definition 4 *(Scientific Workflow). A scientific workflow is a tuple wkf = (cr_w_, SRC_w_, LNK_w_, dsc_w_, dvp_w_, TG_w_), where:*

*• cr_w_ is the credit calculated upon its download times, viewing times, and rating;*
*• SRC_w_ ⊂ SRC is a set of services in wkf;*
*• LNK_w_ ⊂ LNK is a set of data links connecting services in SRC_w_;*
*• dsc_w_ is the text description in the profile of wkf;*
*• dvp_w_ ⊂ DVP is the developer of wkf;*
*• TG_w_ is a set of tags provided by dvp_w_.*

Figure 1D shows a snippet of *S* ^2^ *KG*, which includes several services represented by blue ovals, developers represented by orange ovals, and their relations are represented by arrows with different colors. Specifically, for scientific workflow *BioMart and Emboss Analysis (T2)* in myExperiment, which is a bioinformatic workflow, as shown in Figure 1A, its developer Katy Wolstencroft is represented by orange ovals. Its services are represented by blue ovals; for example, the service *hsapiens_gene_ensembl*. Blue rectangles in wavy rectangles describe the properties of entities, such as the *dmn* and *cr*_*d*_ of Katy Wolstencroft, and the *tl*, *tpc*, *cr*, and *t* of *hsapiens_gene_ensembl*. According to the workflow specification, relations between a developer and his services are extracted as *isDvp* and represented by the orange dotted line; for example, the relation between Katy Wolstencroft and his services *hsapiens_gene_ensembl*. Based on data links in workflows, relations between services are extracted as *isInk* and represented by the gray lines; for example, the relation between the service *hsapiens_gene_ensembl* and the service *getRNorSequence*. Specially, *GetUniqueHomolog* and *CreateFasta* are *beanshells* for cohesion, so they are not regarded as services. Finally, the relation between developers and their friends is extracted as *isFrd* and represented by the yellow arrow in this figure. For example, Katy Wolstencroft, the author of workflows shown in Figures 1A,B, and Alan Williams, the author of the workflow shown in Figure 1C, are friends, and their relation is represented by a yellow arrow and labeled as *isFrd*.

### 2.2 Topic of services

This section constructs topic vectors of services for representing their functions and domains. For a service, the title and text description in its profile prescribe its original functionality. However, since services are constantly being combined for new application scenarios, their profiles can hardly reflect their new application scenarios and functions. As is often the case, various workflow information sharing platforms provide rich descriptions to describe their domains and functions (Gu et al., 2021). Workflows can be regarded as a set of interdependent services that implement complex functions. Based on this observation, we argue that workflows can be considered as the domain of relevant services to provide their integrated functional description. For a more comprehensive representation of service topics, these functions and domains are used to generate topic vectors for the corresponding services. In total, three sample scientific workflows are shown in Figure 1, and they contain similar services but have different descriptions to represent novel domain of services.

Algorithm 1 Service corpus construction

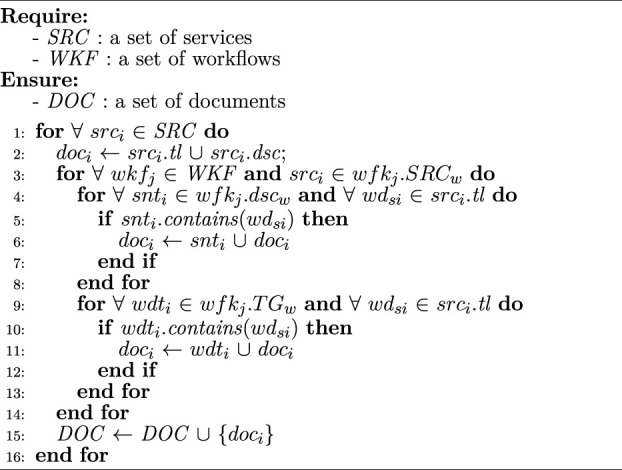

Algorithm 1 presents the construction procedure of service corpus contained in workflows. To prescribe the functionality of each service *src*_*i*_, its title *src*_*i*_.*tl* and text description *src*_*i*_.*dsc* are assembled as a document *doc*_*i*_ (line 2). To present the novel domain of *src*_*i*_, the related description in *wfk*_*j*_.*dsc*_*w*_ and tags in *wfk*_*j*_.*TG*_*w*_ of each workflow *wkf*_*i*_ containing *src*_*i*_ are added to *doc*_*i*_ (lines 3–14), where contains () is a comparison function, *snt*_*i*_ is the *i*th sentence of *wfk*_*i*_.*dsc*_*w*_, *wd*_*si*_ is the *i*th word of *wrc*_*i*_.*tl*, *src*_*i*_.*tl* is the title of *src*_*i*_, and *wdt*_*i*_ is the *i*th tag in *wfk*_*j*_.*TG*_*w*_. All documents construct the corpus for generating topics for services (line 15). Note that *doc*_*i*_ contains several paragraphs, mostly, and could hardly be regarded as a short text, which usually contains less than five words or no more than 140 characters (Li et al., 2016). Therefore, considering the size of *DOC*, the Latent Dirichlet Allocation (LDA) model (Blei et al., 2003) is adopted to generate topics for service corpus. Generally, LDA is a bag-of-words model and widely used in general-scale long text classification, where stop words are removed during the model preprocessing phase.

The time complexity of Algorithm 1 is O (|*SRC*|*|*WKF*|*|*SRC*_*w*_|*|*SNT*|), where |*SRC*| is the number of services in the repository, |*WKF*| is the number of workflows in the repository, |*SRC*_*w*_| is the number of services in the *j*th workflow, and |*SNT*| is the number of sentences in the description of *wkf*_*j*_. Note that line 9 should iterate fewer times than line 4, and thus, the time complexity of Algorithm 1 is determined by lines 1, 3, and 4.

Algorithm 2 Service topic model construction

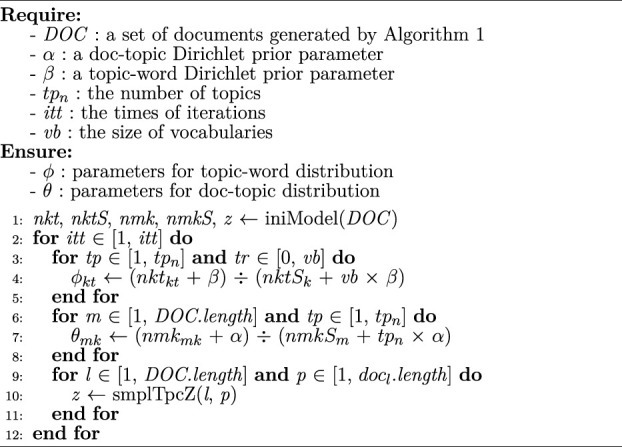

Leveraging *DOC* generated by Algorithm 1, Algorithm 2 introduces the service topic vector construction procedure. Specifically, the model is initialized and parameters are generated leveraging the set of documents *DOC*, where *nkt* is the count of a term for a certain topic, *nmk* is the count of a topic for a certain document, *nktS* is the sum for the *k*th row in *nkt*, *nmkS* is the sum for the *m*th row in *nmk*, and *z* is the generated topic label array (line 1). During each iteration *itt*, we continuously updated the parameters for topic–word distribution *ϕ* (lines 3–5), as well as the parameters for doc–topic distribution *θ* (lines 6–8), where *tp*_*n*_ is the number of topic; *vb* is the vocabulary; *DOC.length* is the size of *DOC*; *doc*_*l*_.*length* is the size of document *doc*_*l*_; and *tp*, *tr*, and *m* are local variables. The Gibbs sampling *smpleTpcZ* () is adopted to update topic label array afterward, where *l* and *p* are local variables (lines 9–11). Please refer to (Blei et al, 2003) for the specific sampling process. The time complexity of Algorithm 2 is O (itt*DOC.length*doc_*l*_.*length*). Note that lines 3 and 6 should iterate fewer times than line 9, and thus the time complexity of Algorithm 2 is determined by lines 2 and 9.

### 2.3 Reputation of services

This section constructs the credit of services through the collective perception of workflows containing these services. A service is applied in multiple workflows with some reputation information representing their popularity. As components of a workflow, the credit of every service contributes to an accurate partial-execution of this workflow, which indicates that users prefer to obtain a service with certain quality. To evaluate the quality of services, the method described in Yao et al. (2014) is used to calculate the credit (*cr*) of services leveraging the workflows information as follows.

Generally, the credit *cr*_*w*_ of a workflow *wkf*_*i*_.*cr*_*w*_ reflects the degree of adoption by developers, and it is calculated by three factors including viewing times (*wkf*_*i*_.*n*_*v*_), download times (*wkf*_*i*_.*n*_*d*_), and rating (*wkf*_*i*_.*n*_*rt*_) by the following formula.

$$wkf_{i}.cr_{w}=f_{\mathrm{crd}}\left(wkf_{i}.n_{v},wkf_{i}.n_{d},wkf_{i}.n_{rt}\right)$$ (1)

Where *f*_*crd*_ is a monotonic increasing function to ensure that the quality of a workflow is directly proportional to its popularity.

For each service in a workflow, its credit can be calculated by adopting a fair-share method, as presented in Nepal et al. (2009). Specifically, due to different importance, the credit of services in a workflow *wkf*_*i*_.*V* should be assigned according to its importance as follows.

$$wkf_{i}.V=v_{1},v_{2},\ldots ,v_{n}$$ (2)

Where *v* ∈ [0, 1] and *∑* *v* = 1. Based on Eq. 2, the credit of each service *src*_*j*_.*cr* is computed using the formula below.

$$\forall src_{j}\in wkf_{i}.SRC_{w}\;src_{j}.cr=v_{j}wkf_{i}.cr_{w}$$ (3)

Since a service *src*_*j*_ may be adopted in several workflows, the average credit is regarded as the credit *src*_*j*_.*cr*.

$$src_{j}.cr=\frac{\sum_{i=1}^{WN}wkf_{i}.src_{j}.cr}{WN}$$ (4)

Where *WN* is the number of workflows containing *src*_*j*_, and *wkf*_*i*_. *src*_*j*_.*cr* is the credit of *src*_*j*_ calculated by *wkf*_*i*_.

### 2.4 Domain and reputation of developers

This section constructs the topic vector of developers for presenting their research domains which influence their services’ and workflows’ functionality and domains. Through examining the information about developers in myExperiment, a developer generally has four features describing his research domains, including his introduction, interests, tags, and field (or industry). These features are adopted to generate topic vectors of corresponding developers leveraging Algorithm 3.

Algorithm 3 Developer topic model construction

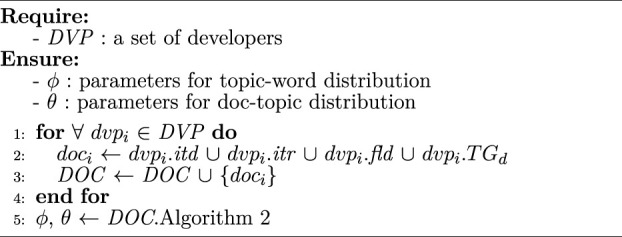

Algorithm 3 shows the construction of topic vectors for developers. To prescribe the domain of each developer *dvp*_*i*_ (line 1), the introduction *dvp*_*i*_.*itd*, interests *dvp*_*i*_.*itr*, field *dvp*_*i*_.*fld*, and tags *dvp*_*i*_.*TG*_*d*_ are assembled into a document *doc*_*i*_ (line 2), and these documents construct the corpus for generating topics of developers (line 3). Specifically, *dvp*_*i*_.*itd* and *dvp*_*i*_.*itr* are texts with several functional paragraphs, and *dvp*_*i*_.*fld* and *dvp*_*i*_.*TG*_*d*_ are some concise words. As mentioned in Section 2.2, *doc*_*i*_ of each developer is not a short text. Thus, the *LDA* model is adopted to generate topic vectors for developers (line 5). Note that the number of iterations in line 1 should be less than that in line 5, so the time complexity of Algorithm 3 is determined by Algorithm 2, where *DOC* is the corpus of developers involved in this algorithm.

The reputation is calculated to reflect the trust degree of a developer. We use the method proposed in Yao et al. (2014) to calculate this value using the developer’s rating and his services’ credit. Specifically, each developer in myExperiment has an average rating to reflect his contribution. Hence, the rating is considered as an important feature for calculating the reputation. In addition, the credit of his previously developed services is another feature that indicates his reputation. Therefore, the reputation *cr*_*d*_ of a developer is calculated leveraging the follow formula (Yao et al., 2014).

$$cr_{d}=f_{rp}\left(rt_{d_{i}},\left\{p_{d_{i}}\right\}\right)$$ (5)

Where the function *f*_*rp*_ is a monotonic increasing function, which ensures that the reputation of a developer is high when his credit is high and the quality of his services is high as well. $rt_{d_{i}}$ is the rating of a developer calculated by the platform. $\left\{p_{d_{i}}\right\}$ is the credit set of his services.

## 3 Bioinformatic workflow fragment discovery

This section presents the identification of positive and negative links between services to support bioinformatic workflow fragment discovery, involving the selection of candidate atomic services leveraging community detection, and the discovery of their fragments in *S* ^2^KG.

### 3.1 Union impact based on positive and negative links

There exists positive or negative links between pairs of services. Positive links specify correlations, collaborations, and complementary relations between services, whereas on the contrary for negative links. Based on *S* ^2^ *KG*, four types of positive links are identified to guide service cooperation (Ni et al., 2015). A service *src*_*i*_ may compose with another *src*_*j*_, when

1) *src*_*j*_ has a good credit,
2) *src*_*j*_ has a highly similar topic with *src*_*i*_,
3) the developer of *src*_*j*_ is same as that of *src*_*i*_, or
4) the developer of *src*_*j*_ is a friend with similar topics to the developer of *src*_*i*_.

Specifically, the higher the credit of a service is, the higher the possibility that this service is selected to compose a novel workflow. Thus, the positive link *Cr*_*ij*_ between *src*_*i*_ and *src*_*j*_ is calculated to reflect the impact of their credit *src*_*i*_. *cr* and *src*_*j*_.*cr* as follows.

$$Cr_{ij}=src_{i}.cr\times src_{j}.cr$$ (6)

Where *src*_*i*_.*cr* and *src*_*j*_ *cr* are the credit of *src*_*i*_ and *src*_*j*_ calculated by Eq. 4.

• The second positive link *Sim*_*ij*_ is identified to calculate the similarity of *src*_*i*_ and *src*_*j*_ by the following formula Eq. 7 leveraging the services’ topic vectors constructed in Section 2.2.

$$Sim_{ij}=\frac{\sum_{k=1}^{tp_{n}}\left(src_{i}.tpc_{k}\times src_{j}.tpc_{k}\right)}{\sqrt{\sum_{k=1}^{tp_{n}}{\left(src_{i}.tpc_{k}\right)}^{2}}\times \sqrt{\sum_{k=1}^{tp_{n}}{\left(src_{j}.tpc_{k}\right)}^{2}}}$$ (7)

Where *src*_*i*_.*tpc*_*k*_ and *src*_*j*_.*tpc*_*k*_ are the values of the *k*th feature in *src*_*i*_.*tpc* and *src*_*j*_.*tpc*.*tp*_*n*_ is the total number of topics. The higher the results are, the more similar the two topic vectors are. A threshold *trd*_*t*_ is prescribed to examine whether two services are similar. Intuitively, when *Sim*_*ij*_ ≥ *trd*_*t*_, the topic of two services are similar, and not otherwise.

• The third positive link *Sd*_*ij*_ is identified by Eq. 8 to examine whether the developers of *src*_*i*_ and *src*_*j*_ are same. Considering the stickiness of a developer’s domain, his services should be similar in terms of his topics. These services may be easier to adapt from the perspective of structure, and their composition may match the functional requirements more appropriately.

$$Sd_{ij}=\left\{\begin{array}{cc} 1\; & \text{if}\;\exists \;\left(dvp_{i},\;isDvp,\;src_{j}\right)\\ 0\; & \text{otherwise} \end{array}\right.$$ (8)

Where *dvp*_*i*_ is the developer of *src*_*i*_, and *dvp*_*i*_, *isDvp*, and *src*_*j*_ means that the developer of *src*_*j*_ is also *dvp*_*i*_. As shown in Figure 1A and Figure 1B, these two workflows are constructed by the same developer Katy Wolstencroft. Their structures are similar, but they are adopted in different domains and have different titles and introductions.

• The fourth positive link *Sf*_*ij*_ is identified, when two developers are friends, their domains and interests may be similar. Thus, the topic of services they developed should be similar.

$$Sf_{ij}=\left\{\begin{array}{cc} 1\; & \text{if}\;\exists \;\left({dvp}_{i},\;isFrd,\;{dvp}_{j}\right)\\ 0\; & \text{otherwise} \end{array}\right.$$ (9)

Where (*dvp*_*i*_, *isFrd*, and *dvp*_*j*_) means that the developer *dvp*_*i*_ of *src*_*i*_ is a friend of the developer *dvp*_*j*_ of *src*_*j*_. As shown in Figure 1, the developers of Figure 1B and Figure 1C are friends. As we can see, they have constructed the similar workflows with the same title and different functional description.

Negative links indicate functionality uncorrelations, conflicts, or even competitions between services. Based on *S* ^2^ *KG*, a union negative link *TC*_*ij*_ is identified leveraging *Cr*_*ij*_ and *Tm*_*ij*_. Specifically, *Tm*_*ij*_ is a negative link specifying that services may cooperate with very low feasibility if they have not cooperated in the same workflow since their creation. *Tm*_*ij*_ is calculated as follows.

$$Tm_{ij}=now-\max\left(src_{i}.t,src_{j}.t\right)$$ (10)

Where now is the current time. The uncooperative duration of two services is determined by the latest service. The larger the value of *Tm*_*ij*_ is, the less likely that these two services are cooperated to construct a novel workflow.

Based on *Tm*_*ij*_, *TC*_*ij*_ can be formed as follows to present that two services are unlikely to cooperate.

$$TC_{ij}=Tm_{ij}\times Cr_{ij}$$ (11)

Generally, the larger the value of *TC*_*ij*_ is, the lower the feasibility that *src*_*i*_ and *src*_*j*_ can be cooperated.

As mentioned before, given two services, we adopted the union impact *U*_*ij*_ through integrating positive and negative links to determine whether they can be cooperated, as follows.

$$\begin{array}{c} U_{ij}=\alpha \times Sim_{ij}+\beta \times Cr_{ij}-\gamma \times TC_{ij},\\ if\;{Sd}_{ij}=1\;or\;{Sf}_{ij}=1 \end{array}$$ (12)

Where *α*, *β*, and *γ* are the importance of each influencing factor, and *α* + *β* + *γ* = 1.

### 3.2 Service discovery leveraging community detection

Due to the different levels of users’ expertise, a requirement in this study is composed of several sub-requirement descriptions in an effort to express the requirement more clearly. Generally, it can be formalized in terms of *Q* = {*q*_1_, *q*_2_, …, *q*_*m*_}. For each sub-requirement, an appropriate service is discovered accordingly. To facilitate single service discovery from the functional perspective, services and *isInk* relations are extracted from *S* ^2^ *KG* and construct a Service Invoking Network (*SINet*). For example, the service *hsapiens_gene_ensembl* and the service *getRNorSequence* in the workflow *BioMart and Emboss Analysis (T2)* are divided into the same purple community because of similar application scenarios. The fast unfolding method (Blondel et al., 2008), which is heuristic based on modularity optimization, is adopted to divide *SINet* into several functional communities. This method adjusts the division of communities by continuously optimizing the modularity, where the modularity of a partition is a measure of the density of links within the community and the density of links between communities (Newman, 2006) as defined by Eq. 13.

$$CM=\frac{1}{2m}\times \underset{i,j}{\sum }\left[A_{ij}-\frac{k_{i}k_{j}}{2m}\right]\times \delta \left(c_{i},c_{j}\right)$$ (13)

$$k_{i}=\underset{j}{\sum }A_{ij}$$ (14)

$$m=\frac{1}{2}\underset{i,j}{\sum }A_{ij}$$ (15)

$$\delta \left(c_{i},c_{j}\right)=\left\{\begin{array}{c} 1\;c_{i}=c_{j}\;\\ 0\;others\; \end{array}\right.$$ (16)

Where *A*_*ij*_ represents the wight of the link between *src*_*i*_ and *src*_*j*_, and the wight is *Sim*_*ij*_ as calculated by Eq. 7. *k*_*i*_ is the sum of wights of links which connect to *src*_*i*_. *m* is the sum of link wights in SINet. *c*_*i*_ is the community to which *src*_*i*_ is assigned. *δ*(*c*_*i*_, *c*_*j*_) represents the fact that whether *c*_*i*_ and *c*_*j*_ are same. By dividing SINet into communities, the entire network is a set *CM* of communities, and each community *c*_*i*_ in *CM* is a tuple *c*_*i*_ = {*c*_*t*_, *CS*}, where *c*_*t*_ is the topic vector of the representative service of *c*_*i*_ and *CS* is a set of services in *c*_*i*_.

Algorithm 4 Candidate service discovery

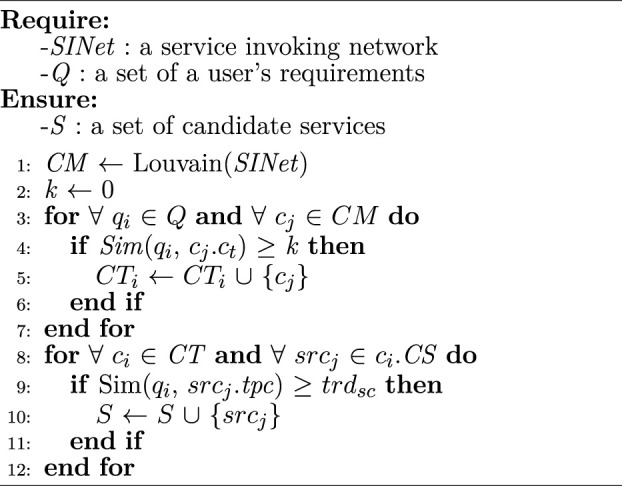

Based on *CM*, the most relevant communities and candidate services are discovered. Algorithm 4 represents candidate communities and services discovery procedure. First, SINet is divided into several communities leveraging the fast unfolding method (Blondel et al., 2008) according to service topics (line 1). A comparison variable *k* is set to 0 (line 2). For each sub-requirement *q*_*i*_ and each community *c*_*j*_, the functional similarity between them is calculated by the comparison function *Sim*() and compared with *k*, where *q*_*i*_ is vectorized by embedding. The community *c*_*j*_ with the most similar functionality to *q*_*i*_ is inserted into a set of candidate communities *CT* (lines 3–7). For each candidate community in *CT*, the similarity of each *src*_*j*_.*tpc* and *q*_*i*_ is calculated and compared with the pre-specified threshold *trd*_*sc*_. If the similarity is larger than *trd*_*sc*_, *src*_*j*_ is inserted into the set *S* as candidate services (lines 8–12). The time complexity of Algorithm 4 is O (|*CT*|*|*CS*|), where |*CT*| is the number of *CT* and |*CS*| is the number of services in the community *c*_*i*_. Note that line 3 should iterate fewer times than line 8, and thus, the time complexity of Algorithm 4 is determined by line 8.

### 3.3 Bioinformatic workflow fragment discovery

Based on candidate services discovered by Algorithm 4, this section proposes to discover appropriate workflow fragments, where relations prescribed by *S* ^2^KG are obtained to connect candidate services for respective service stubs in the requirement. The Yen’s method (Yen, 1971), which is a heuristic method widely used in graph traversal, is adopted to discover and compose relevant workflow fragments from various workflows.

Algorithm 5 CFDY: Crossing-workflow fragment discovery using Yen’s method

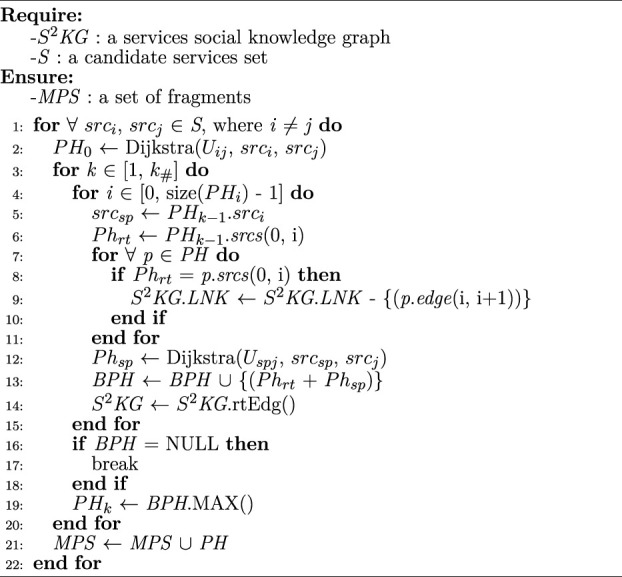

The Algorithm 5 (denoted *CFDY*) shows the procedure of discovering appropriate bioinformatic workflow fragments. First, the *Dijkstra* () is adopted to find the optimal combinatorial fragment *PH*_0_ from the service *src*_*i*_ to the service *src*_*j*_ leveraging the union impact *U*_*ij*_ (lines 1,2). Based on *PH*_0_, the *k*th combinatorial fragment is found (lines 3–19). Above all, every deviated service is traversed (lines 4–15). Specifically, *src*_*sp*_ is retrieved from the (*k-1*)th best combinatorial fragment and *Ph*_*rt*_ records the sequence of services from *src*_*i*_ to *src*_*sp*_ (lines 4–6). The links that belong to part of the previous best combinatorial fragment of the same *Ph*_*rt*_ are removed from the *S* ^2^ *KG* (lines 7–11). The combinatorial fragment from *src*_*sp*_ to *src*_*j*_ is found by the *Dijkstra* () and recorded to *Ph*_*sp*_ (line 12). Entire combinatorial fragment is made up of *Ph*_*rt*_ and *Ph*_*sp*_ and added to the set *BPH* (line 13). The links that were removed before are added back to *S* ^2^ *KG* (line 14). If there are no other combinatorial fragments, the method ends (lines 16–18). The optimal combinatorial fragment in *BPH* is the *k*th combinatorial fragment *PH*_*k*_ (line 19). All paths in *PH* are added to the set *MPS* (line 21). The time complexity of Algorithm 5 is O (|*S*|*k_#_*size (*PH*)*|*PH*|), where |*S*| is the number of candidate services, *size(PH)* is the value of *size* (*PH*_*i*_) minus 1, and |*PH*| is the number of path.

## 4 Implementation and evaluation

This section presents our experiments and evaluation results. Experiments are performed on a desktop computer with an Intel i7 6,700 processor at 3.40 and 3.41 GHz, 8.00 GB of RAM and a 64-bit Windows 10 operating system. The prototype is implemented by *Python* and Java.

### 4.1 Data set and preprocessing

This study adopts bioinformatic workflows in *myExperiment* for our experiments, where workflows in the *Taverna 2* category by May 2019 are crawled. For each service, its title, description, created time and developer are collected. For each workflow, its title, description, tags, publishing date, download times, viewing times, rating, developer, services and data links are collected, where the data links reflect the control flows between services (i.e., invocation relations). For each developer, his name, introduction, interests, field, rating and friends are collected. Note that services and workflows without a title or description are deleted. As a summary, the numbers of available services, workflows, developers and their relations are shown in Table 1.

**TABLE 1:** Data set in *Taverna 2*.

Statistics	Value
# of service	2,870
# of workflow	1,058
# of developer	175
# of *isInk*	2,516
# of *isDvp*	2,870
# of *isFrd*	271

The data cleaning procedure is conducted, where stop words are removed, and the stemming of words is extracted. Thereafter, entities and relationships are extracted, and their attributes are obtained by the techniques presented in Section 2. We adopt the graph database Neo4j (Robinson et al., 2015) to store these cleaned data.

To evaluate the efficiency of our technique, we have generated 40 crossing-workflow fragments leveraging legacy workflows as testing fragments based on *S* ^2^ *KG*. According to the statistic reported in our previous work (Zhou et al., 2020), roughly 86% of workflows contains no more than 11 services. Therefore, 5 out of 40 testing fragments are set to contain over 11 services.

### 4.2 Measurement metrics

Three metrics are adopted to evaluate the accuracy and effectiveness of our technique as follows:

• *P*: The precision (denoted *P*) indicates the percentage of the number of correctly recommended services over the total number of recommended services.

$$P=\frac{\left|CS_{pt}\cap CS_{rc}\right|}{\left|CS_{rc}\right|}$$ (17)

• *R*: The recall (denoted *R*) refers to the percentage of the number of correctly recommended services over the total number of desired services.

$$R=\frac{\left|CS_{pt}\cap CS_{rc}\right|}{\left|CS_{pt}\right|}$$ (18)

• *F1*: The *F1* score is used for an overall evaluation based on *P* and *R.*

$$F1=\frac{2\times P\times R}{P+R}$$ (19)

Where *CS*_*pt*_ is the expected service set, and *CS*_*rc*_ is the set of recommended services.

### 4.3 Baseline techniques

In this section, the following four state-of-the-art techniques are chosen as baselines to evaluate the effectiveness of our technique:

• C*SBR* (Gu et al., 2021) is a semantics-based model to compose and recommend a set of complementary services for workflow construction. By applying this approach, we first construct a semantic service bundle repository using experimental data. Then a bundle of complementary services is recommended to fulfill the sophisticated requirements. Finally, a more suitable result is found using a greedy approximation method considering the time complexity.
• *ClstRec* (Conforti et al., 2016) is a modularized clustering algorithm to generate service clusters. We first identify target clusters for each service stub, find their services or fragments therein, and sort them into candidate services or fragments. Then a series of fragments are constructed across workflows based on their relations. Based on their similarity, these fragments are identified, ranked, and recommended accordingly.
• *CDSR* (Xia et al., 2015) is a category-aware clustering and distributed service recommending method to automatically create fragments. First, we cluster the experimental data into various categories based on the similarity of functionality and popularity of their services. Then we map requirements to relevant categories to find candidate services. Finally, these candidate services in the most relevant categories construct cross-workflow fragments to fulfill the requirements.
• *Short Path* (denoted *SP*) method is a classical heuristic algorithm. First, we start to navigate from a service and select the neighbors with the highest relevance, which have a connection-aware relation with it, according to a given probability distribution. Then a similar operation is performed starting from that service to find a service fragment.

### 4.4 Evaluation results

In this section, we first optimize the algorithm *CFDY* by adjusting the following parameters *tp*_*n*_ and *k*_*#*_ and then use the parameters *sq*_*#*_ and *trd*_*U*_ to discuss the evaluation results of *CFDY* and baselines.

• *tp*_*n*_: *The topic number.* The semantic description is susceptible to the topic number. Different number of topics should lead to different partitions of services and developers and recommend various results. Therefore, determining an appropriate *tp*_*n*_ is fundamental and crucial.
• *k*_*#*_: *The number of paths.* When it changes, it should affect the number of path searches of *CFDY.* Different *k*_*#*_ should affect the number of services in results, thereby affecting the efficiency of *CFDY.*
• *sq*_*#*_: *The number of sub-requirements.* With the increase of *sq*_*#*_, the number of service and the complexity of fragments should increase, thereby affecting the efficiency of the fragment discovery.
• *trd*_*U*_: *The connection-aware threshold of two services.* It should influence the efficiency of our method by changing the scale of candidate services set.

#### 4.4.1 Impact of *tp*_*n*_

To select the optimal *tp*_*n*_, a widespread perplexity is used to calculate the quality of the LDA model, as shown below, which describes the degree of uncertainty of the model about documents and their topic. Therefore, the lower the perplexity is, the better predictive effect.

$$\text{Prp}=\exp\left\{-\frac{\sum_{d=1}^{M}\log p\left(w_{d}\right)}{\sum_{d=1}^{M}N_{d}}\right\}$$ (20)

Where *M* is the number of *DOC*, *N*_*d*_ represents the number of words in a *doc*, and *p* (*w*_*d*_) is the probability of that the word *w*_*d*_ is contained in the *doc*.

As shown in Table 2, the perplexities of services’ and developers’ LDA models are calculated separately. For the LDA model of services, with the increasing of topic number (denoted as *tp*_*ns*_), its perplexity (denoted as *Prp*_*s*_) decreases. When *Prp*_*s*_ is 131.505, *tp*_*ns*_ is selected to the optimal value as 43. For the LDA model of developers, its perplexities (denoted as *Prp*_*d*_) are calculated when its topic number (denoted as *tp*_*nd*_) ranges from 2 to 13. When *tp*_*nd*_ is 10, *Prp*_*d*_ is the smallest value as 454.085. Therefore, 43 and 10 are determined as the *tp*_*n*_ of two LDA models.

**TABLE 2:** *Prp* settings with various *tp*_*n*_.

*tp*_*ns*_	10	20	30	40	42	43	44	45	46
*Prp*_*s*_	196.860	149.406	135.332	132.726	132.176	131.505	131.757	132.750	132.752

*tp*_*nd*_	2	4	6	8	9	10	11	12	13
*Prp*_*d*_	654.289	509.672	468.705	468.485	465.883	454.085	463.771	479.020	479.157

#### 4.4.2 Impact of *k*_*#*_

**FIGURE 2:**
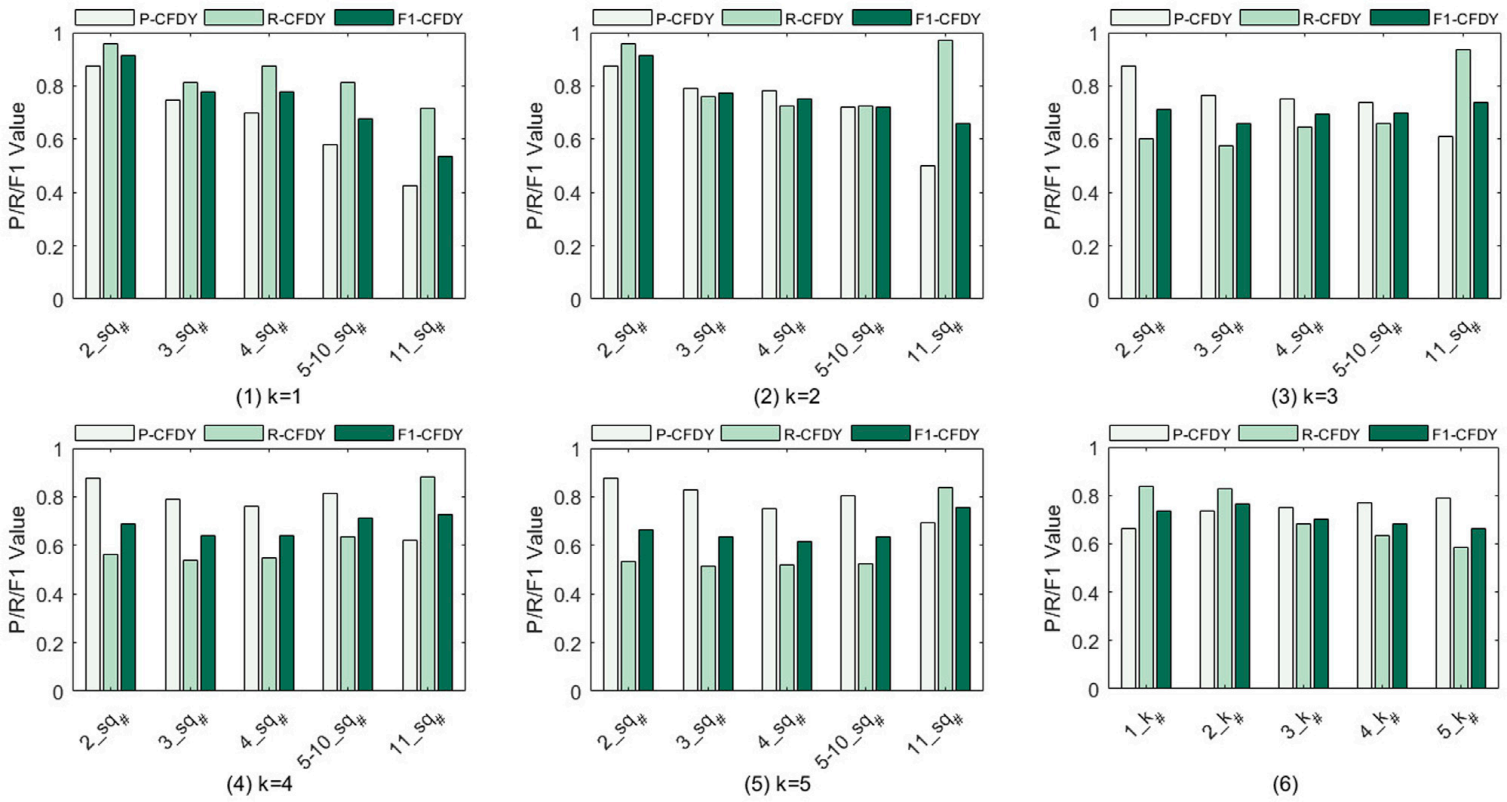
Precision, recall, and F1 value for the fragment discovery when *k*_*#*_ is set to 1, 2, 3, 4, and 5, respectively.

The influence of different *k*_*#*_ on *P*, *R*, and *F1* is shown in Figure 2 when *k*_*#*_ is set to 1, 2, 3, 4 and 5, respectively. *tp*_*ns*_ is set to 43, *tp*_*nd*_ is set to 10, and *trd*_*U*_ is set to 0.8. Considering that different *sq*_*#*_ will affect the complexity of fragments discovery, the efficiency of various *k*_*#*_ is evaluated with test examples containing 2–11 sub-requirements. Considering that the number of test examples containing 5–10 sub-requirements is small, these examples are grouped into one category.

• Figure 2(1) and Figure 2(2) show that, as *sq*_*#*_ gradually increases, *P*, *R* and *F1* as a whole gradually decrease. In particular, in Figure 2(2), when *sq*_*#*_ is 11, there is a sudden change in *R* that has a higher value. The increase of *sq*_*#*_ leads to the increase of the number of services contained in *CS*_*pt*_. Therefore, the fragment structure becomes more complex. Generally, when *sq*_*#*_ is larger, the single path search can hardly fulfill the complexity requirement well. Compared with Figure 2(1), our technique increases the number of paths, and the number of services contained in *CS*_*rc*_. Thus, *R* is increased to some extent.
• As shown in Figure 2(3)–2 (5), when *k*_*#*_ is larger than 2, their *P*, *R* and *F1* have similar tends. With the gradual increase in *sq*_*#*_, *P* and *F1* gradually decrease, but *R* has increased to a certain extent. In the same way, the decrease in *P* and *F1* is due to the increase in *sq*_*#*_ and the complexity of their structure. With the increase of *k*_*#*_, the number of services in *CS*_*rc*_ increases, which will lead to an increase in *R.* However, as *k*_*#*_ gets larger, too much exploration will lead to a decrease in *R* when *sq*_*#*_ is small. For example, compared with *R* in Figure 2(1), *R* in Figure 2(5) is significantly lower when *k*_*#*_ equal to 2.
• Figure 2(6) shows the average values of *P*, *R*, and *F1*, at each *k*_*#*_. Larger *k*_*#*_ means that more fragments are constructed. Due to the increase of fragments, more services are selected. Hence, this result may lead to an increase of *P.* However, since the number of expected services is fixed, blindly increasing the number of recommended services by adding too many paths may not maintain the increase in *R. F1* has a maximum value at 2. Therefore, 2 is finally selected as the value of *k*_*#*_ in subsequent experiments.

#### 4.4.3 Comparison on *sq*_*#*_

**FIGURE 3:**
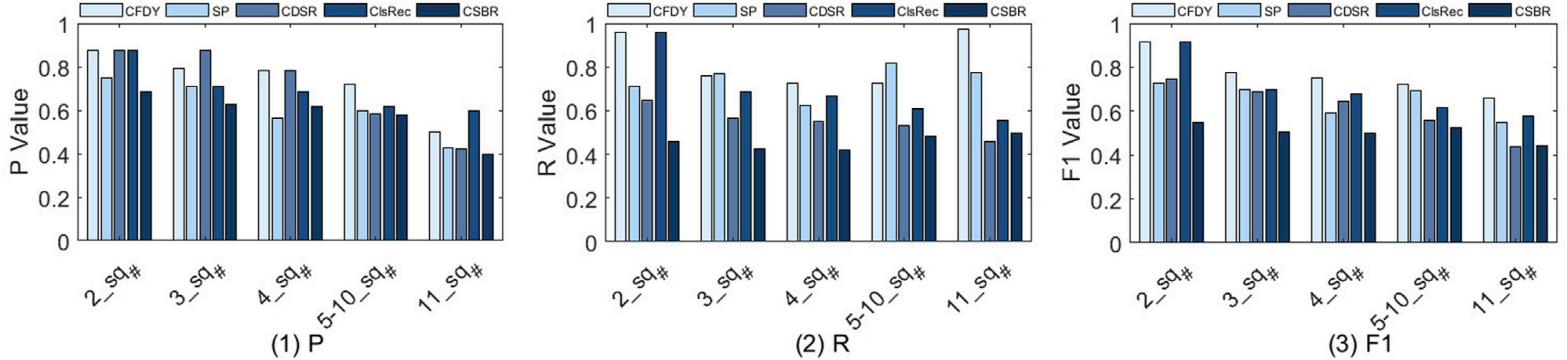
*P*, *R*, and *F1* value for the fragment discovery, when the sub-requirements number is set to 2, 3, 4, 5–10 and 11, respectively.

We compare five methods and estimate the influence of different *sq*_*#*_ for fragment discovery. The results of *P*, *R*, and *F1* are shown in Figure 3 when *sq*_*#*_ is set to 2, 3, 4, 5–10, and 11, respectively. *tp*_*ns*_ is set to 43, *tp*_*nd*_ is set to 10, *k*_*#*_ is set to 2, and *trd*_*U*_ is set to 0.8.

Figure 3(1) shows that *P* decreases with the increase of *sq*_*#*_, and the overall performance of *CFDY* is better than other methods.

• For CFDY, as *sq*_*#*_ increases, its p decreases. When *sq*_*#*_ is small, its structure is relatively simple, and the expected service can be selected more accurately by using the relationship between services. With the increase of *sq*_*#*_, the structure of the fragment becomes more complex and contains more branches. Therefore, the selection of candidate services brings certain difficulties, and thus the accuracy rate is reduced.
• Compared with *CFDY*, *SP* only explores a single path and lacks the exploration of branches. Its recommended fragment does not contain some of the expected services present in the branch. As a result, the number of expected services in its recommendation set is less than *CFDY*, which results in its *P* being lower than that of *CFDY.*
• In fact, *CSBR* pursues more semantic similarity matching, and the consideration of structural similarity is not a priority, which leads to the fact that most of the services it recommends are not the expected ones. Therefore, its overall performance is the lowest compared to others.
• *CDSR* uses category awareness to cluster services and considers the impact of service coexistence time on its relationship when considering historical combination information. By considering the functional similarity and relations, when *sq*_*#*_ is less than 5, its *P* is relatively high. But when *sq*_*#*_ is too large and the fragment structure is too complex, its consideration of semantics and structure can hardly fulfill the requirement.
• Similarly, *ClstRec* uses the description of services to cluster them, and selects candidate services from suitable clusters for each sub-requirement. However, this method does not pay too much attention to structural information and can hardly guarantee the rationality of service composition. This causes *P* to be lower when *sq*_*#*_ is large and the fragment structure is more complex.

Figure 3(2) shows *R* of five methods. Overall, the *R* of *CFDY* is higher than that of other methods.

• For *CFDY*, as *sq*_*#*_ increases, its *R* first decreases and then increases. On the whole, its *R* is the highest compared to other methods. When *sq*_*#*_ is small, its structure is relatively simple, and too many exploration branches will add some unexpected services to the recommended fragment. Therefore, its *R* decreases. As *sq*_*#*_ becomes larger, the structure of the fragment becomes more complex and contains more branches. Therefore, further exploration of branches will increase *R* to some extent.
• Similarly, since *CSBR* lacks consideration of structural similarity, most of the services contained in its recommended fragment are unexpected, so its *R* is the lowest.
• Since *SP* has not further explored branches, its *R* is overall lower than that of *CFDY.* When *sq*_*#*_ is at 5–10, its *R* is higher than that of *CFDY.* Because it only explores a single path, in the case of more branches, the number of services in the fragment it recommends is much smaller than *CFDY*, which causes its *R* to be higher.
• For *ClsRec*, when *sq*_*#*_ is smaller, it has a higher *R.* Because it focuses on the similarity of functions, when *sq*_*#*_ is small and the fragment structure is simple, it can relatively accurately find expected services. However, when the fragment becomes complicated, this method can hardly effectively find all expected functions, due to the lack of comparison of structural similarity.
• *CDSR* first finds candidate services according to functional category, then uses historical usages and the coexistence time of services to construct fragments. This method considers the structure of fragments to a certain extent, but can hardly guarantee the necessity of the recommended services. Therefore, the recommended fragments contain a large number of unexpected services, which leads to a lower *R.*

As shown in Figure 3(3), the *F1* of five method decreases as *sq*_*#*_ increases.

• Compared with other methods, *CFDY* has the highest *F1.* Because it considers the structural similarity of fragments while considering semantic similarity. As the *sq*_*#*_ increases, the requirement of a user becomes more and more sophisticated, which leads to more complex selection of candidate services, and more complex fragment discovery and recommendation. Its *F1* is the highest when *sq*_*#*_ is 2, indicating that its recommendation effect is the best when the recommended fragment contains two services and their relationships. This value does not reach 1, because the function descriptions of some services are too similar, resulting in too high similarity of their topic vectors, so that they can hardly be accurately distinguished when selecting candidate services. This is an inevitable problem of *LDA* model.
• For *SP*, due to its low applicability when the fragment structure is complex, its *F1* is lower than that of *CFDY.* The other three methods divide services into different categories, clusters or packages according to their functions, and use semantic similarity to select candidate services. They lack the comparison of structural similarity. In contrast, both *CDSR* and *ClsRec* use historical relations between services to calculate the similarity in fragment structure, while *CSBR* only considers the feasibility of combinations in terms of functional similarity, which leads to the lowest *F1.*

#### 4.4.4 Comparison on *trd*_*U*_

**FIGURE 4:**
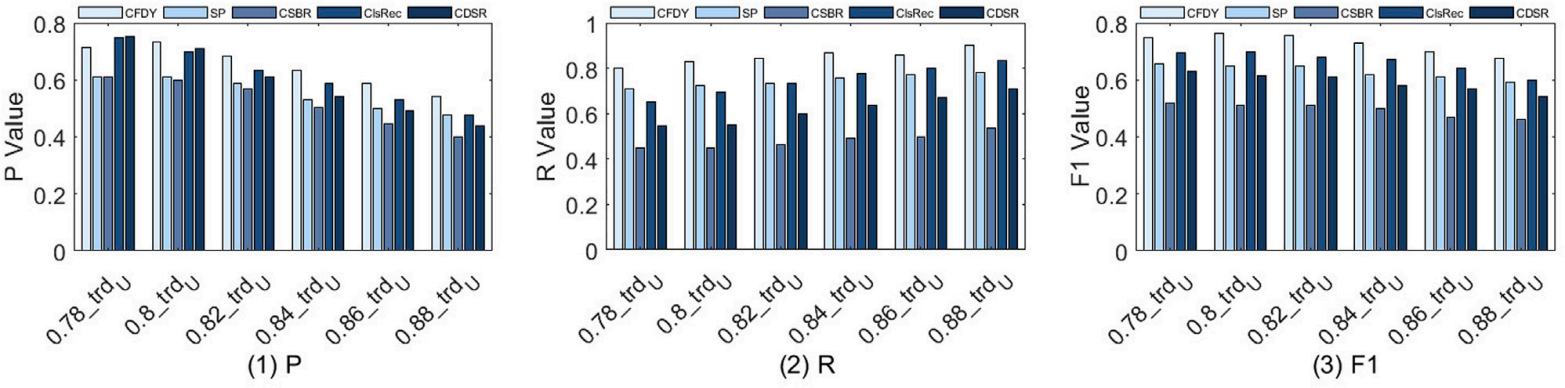
*P*, *R*, and *F1* value for the fragment discovery when the connection-aware threshold is set to 0.78, 0.80, 0.82, 0.84, 0.86, and 0.88, respectively.

We estimate the influence of different *trd*_*U*_ for bioinformatic workflow fragment discovery and the results of *P*, *R* and *F1* are shown in Figure 4 when *trd*_*U*_ is set to 0.78, 0.80, 0.82, 0.84, 0.86 and 0.88, respectively. *tp*_*ns*_ is set to 43, *tp*_*nd*_ is set to 10 and *k*_*#*_ is set to 2. Since the test set contains various samples with different *sq*_*#*_, the final result is the average of all test results.

The results in Figure 4(1) show that *P* of *CFDY* is the highest overall compared to other methods.

• Similarly, when semantic similarity is considered, *CFDY* has more exploration branches compared to *SP*, so the recommended fragment contains more expected services. Specifically, when *trd*_*U*_ is higher, the number of candidate services that can be selected decreases, and the number of expected services that are missing in the recommended fragment increases. This results in *P* getting smaller and smaller as *trd*_*U*_ increases. Especially for *CFDY*, *P* at 0.8 is greater than that at 0.78, which is caused by the uneven distribution of service in *S* ^2^ *KG* and the large difference in in-degree and out-degree of them.
• Since *CSBR* doesn’t consider the structural similarity much, its *P* is the lowest among all methods. It only relies on the functional similarity between services to discover a crossing-workflow fragment. When *trd*_*U*_ is higher, the number of candidate services for selection decreases, which affects its recommendation effect. Compared with *CSBR*, although *SP* considers the similarity of the fragment structure to a certain extent, it does not further explore branches and its accuracy is only higher than that of *CSBR.* Compared with the above two methods, *ClsRec* and *CDSR* have higher *P.* Generally, they adopt the clustering and classification to compare the functional similarity of fragments, and also apply historical usages to evaluate the structural similarity of fragments. Therefore, they are more effective than the methods that only consider semantic similarity. However, lacking the exploration of social relations, they are not as effective as *CFDY.*

Figure 4(2) shows the comparison of *R*. Similarly, *R* of *CFDY* is the highest, while *R* of *CSBR* is much lower than the other four methods. The difference is that, compared with *P*, as *trd*_*U*_ increases, the *R* of five methods increases.

• As the threshold increases, the candidate services become more similar and these services are more likely to be expected services. Some unexpected services are filtered out and the expected services are more likely to be included in the recommended fragment. For *CFDY*, the variation of *trd*_*U*_ affects the selection of its candidate services. However, compared with other methods, the consideration of social information on the discovery of crossing-workflow fragments can ensure the functional similarity and structural rationality of fragments to a certain extent and a better effect can be obtained.
• For *CSBR*, since it pursues more semantic similarity without considering the fragment structure, and does not consider the structural matching between services, its recommended fragment contains more unexpected services than other methods, which leads to its *R* is the lowest. *CDSR* uses historical usages information to ensure the rationality cross-workflow fragment structure. As a result, the recommended fragment contains a relatively high number of expected services, which results in a higher *R* than that of *CSBR.*
• Similarly, because *SP* and *ClsRec* add the similarity evaluation of the recommended cross-workflow fragment structure, their *R* are higher than that of *CSBR.* The difference in the recommended effects of *SP*, *ClsRec* and *CDSR* is caused by their different calculation methods of functional similarity. In addition, the fragment complexity recommended by *SP* is lower than other methods and its fragment contains a relatively small number of services, which is part of the reason for its high *R.*

Finally, the comparison results of *F1* of the five methods are shown in Figure 4(3). This figure shows that the *F1* of each method decreases according to the changes of *P* and *R*. In general, *CFDY* has the highest *F1* and *CSBR* has the lowest one.

• For *CFDY*, as the *trd*_*U*_ increases, there are fewer connections that can meet the requirements, which leads to some feasible solutions to be ignored, thereby reducing *P.* At the same time, the reduction of the candidate set can increase the possibility of selecting the expected services, so that *R* increases. However, in combination, the increase in *R* is less than the decrease in *P*, so *F1* decreases. Since the *P* of *CFDY* at 0.8 is greater than that at 0.78, the *F1* of *CFDY* at 0.80 is higher than that at 0.78.
• Due to the lack of comparison of structural similarity in *CSBR*, its *F1* is the lowest. It shows that structural similarity is an important factor in cross-workflow fragment discovery and recommendation. Blindly pursuing functional similarity while ignoring structural similarity cannot achieve better recommendation results. Compared with *CSBR*, the other three methods leverage some structural information, thereby obtaining better *F1.* But compared with *CFDY*, they lack the exploration of the social relations between services, so *F1* is lower.

A higher *F1* of *CFDY* indicates that reasonable social information can improve the effectiveness of cross-workflow fragment discovery and recommendation to some extent. In fact, the representation of the functional domain of a service can be enhanced by using social information. In addition, author information can be used to reveal the hidden relationships between services. Therefore, it has a positive impact on fragment discovery and recommendation.

## 5 Related works

### 5.1 Social-aware workflow fragment discovery

Workflow fragment recommendation is an important research problem in the field of service computing (Coleman et al., 2022). It can shorten development cycles and reduce the cost by recommending suitable services and workflow fragments for users (Almarimi et al., 2019) from an open, large-scale library of Web services (Modi and Garg, 2019). In the past, profiles of services and workflows were used as the only guide for users to discover workflow fragments. However, with the development of social network service (SNS), traditional service repositories have become increasingly social, and contain a wealth of social information reflecting the social connections of developers and services (Bastami et al., 2019). This social information can also have an impact on workflow fragment recommendation, whereas existing approaches did not take full advantage of this complex social information currently.

Authors (Gu et al., 2021) propose a service package recommendation model (*CSBR*) based on a semantic service package repository by mining existing workflows. Using the degree of service co-occurrence, the correlation between service and workflow is mined. Specifically, reusable service packages composed of multiple collaborative services are annotated with composite semantics instead of their original semantics. Based on the semantic service pack repository, *CSBR* can recommend service packs that cover the functional requirements of workflow fragments as completely as possible. However, this approach discusses only some social properties and lacks further exploration of social relations, making it difficult to reveal the implicit relations between services.

Xia et al. (2015) used the categories of services to construct workflow fragments. They propose a category-aware distributed service recommendation (CDSR) model based on a distributed machine learning framework. Experiments on real data sets prove that the proposed method not only achieves a significant improvement in accuracy, but also enhances the diversity of recommendation results. However, this method ignores the relations between services and can hardly guarantee the structural similarity of the recommended workflow fragments.

Yao et al. (2014) proposed a ReputationNet to facilitate the workflow fragment discovery. Based on the ReputationNet, the reputations of services and its developers are calculated and represented. According to this, the services and workflows that have better qualities can be recommended to users to satisfy their sophisticated and complicated business requirements. This method utilizes the social attribute reputation, which can improve the efficiency of fragment recommendation to a certain extent. However, many other social information, such as social relations which can promote users to mine latent knowledge, have not been considered.

Zhu et al. (2021) proposed a new model SRaSLR, which is a type of social-aware service label recommendation model. There are invocation and dependency relations between services, and these relations make services naturally constitute a service social network. The authors combine the textual information in service profiles and the social network relations between services. Based on the feature fusion of two perspectives, a model based on deep learning is constructed. Authors conduct a lot of experiments on real-world Programmable Web data set, and the experimental results show that the use of social relations can improve the performance of recommendation.

Khelloufi et al. (2021) argued that combining users’ social characteristics can improve the efficiency of services recommendation and help us provide context-aware services. Therefore, they exploit the social relationships defined in SIoT to build service recommendations among devices, and thus, to enhance service discovery and composition. They propose a SIoT-based service recommendation framework in which devices inherit social relationships from their owners to provide socially aware service recommendations. A boundary-based community detection algorithm is proposed to form a community of socially connected devices.

Kalaï et al. (2018) adopted the social information about the users and the profiles about services to build a social-aware graph for services recommendation. The widespread use of social media provides a large amount of social information for service repositories. Using social information, many user relationships can be extracted for capturing implicit relationships between services. For example, two users, who are friends with each other, may be interested in similar service features. Based on the interests of a user and his friends, personal service recommendations can be provided. However, workflow fragments that can accomplish complex requirements may be preferable to users than recommending a single service that can accomplish simple and specific tasks for them.

Liang et al. (2016) proposed a new framework to effectively discover appropriate services by combining social media information. Specifically, they propose different methods to measure the four social factors collected from Twitter that semantic similarity, popularity, activity and decay factors. Qiao et al. (2019) proposed a recommendation algorithm based on knowledge graph representation learning, which embeds the entities and relations of knowledge graph into a low-dimensional vector space. These methods consider some social attributes in service recommendation, reflecting the importance of social information in recommendation work. However, they mainly recommend a single service to users, and can hardly be used to discover workflow fragments to fulfill the complex requirements prescribed by certain users.

Based on the various types of data in service repositories, underlying logical relations among them can be found to facilitate workflow fragment discovery and recommendation (Wang et al., 2019). Authors propose a fine-grained knowledge graph (DUSKG) to represent the information about users, services and service value feature (VF) and their relations. Based on the DUSKG, the VFs that a service has, the VFs which a user is interested in, and the relations between users and services can be expressed intuitively. Leveraging the DUSKG, five methods are adopted to recommend reasonable single services. However, this method also ignores the importance of workflows which can accomplish complex tasks.

### 5.2 Semantics-based workflow fragment discovery

Techniques have been developed to recommend workflow fragments from a functional perspective (Hao et al., 2019). Conforti et al. (2016) proposed a technique for automatically discovering hierarchical workflow fragments containing interrupted and non-interrupted boundary services markers. This technique uses approximate functions and contains dependency discovery techniques to extract the process-subprocess hierarchy. Profiles and service invocation relations are used for workflow fragment discovery. However, this method has not yet considered the social information that has an impact on the workflow fragment recommendation, and the information in the repository is not considered comprehensively.

Since the profiles of services are static and the development process is iterative (Huang et al., 2012), Modi and Garg (2019) proposed a method to update the profile of a single service leveraging the description of related workflows. Supplementary information can update the application scenario of a service and optimize its profile. Using this approach, the accuracy of the functional description of a service can be improved and the available services can be recommended to the user. However due to the limitations of functionality, a single service may not accomplish sophisticated and complicated requirements. Wang et al. (2017) proposed a method to extract fine-grained service value features and distributions for personalization service recommendation. By analyzing comments, the most interest aspects of a user can be learned. According to them, the similarity of these features and the descriptions of services are calculated and services with high similarity will be recommended to the user. However, the application scenarios of a single service are limited, since a single service can hardly satisfy the user’s requirement as well as implement the user’s complex functions completely. This approach lacks to explore the impact of social information and social association on workflow fragment recommendation.

Zhong et al. (2016) and (Hao et al. (2017) extracted the valued information from workflow description to narrow gaps between developers and users. The application scenarios are adopted to supply the description of services to emphasize their functionalities. The *LDA* model is adopted to represent the semantic functions of services. Based on reconstructed descriptions of services, the similarity between services and queries can be improved. However, the similarity is not the only metric that should be considered. Other metrics (Sun et al., 2019), for example, the quality of services (Li et al., 2019), should also be considered in the workflow fragment discovery procedure, so as to guarantee the reliability of the workflow fragments.

Zhou et al. (2018) proposed a method for workflow fragment recommendation which both consider the semantic information of workflows and the hierarchical relations of services. The clustering approach is adopted to cluster the hierarchical structure according to the semantic information, so that the services and workflows with similar functions are in the same group as much as possible. However, this method only considers the invoking relationship between services and does not consider the impact of social connections on workflow fragment recommendation. In fact, these social relations emerge in large numbers in the repositories and also affect the composition of services to a certain extent.

Many services can provide similar functionality, and it is difficult for users to find the service they want (Ren and Wang, 2018). In the workflow fragment recommendation, whether two services can cooperate is an important problem (Lissandrini et al., 2018). The factors that affect service composition usually include two types, positive and negative links. Ni et al. (2015) leveraged tags and both positive and negative links to find service patterns. In addition to positive links of services which facilitate workflows fragment construction, several negative links between services are found, which are strangling service composition. The links between two credible services that have never been cooperated and the links between two services that have been created for a long time but never cooperated together are negative links. Although the consideration of negative links can guide whether two services can be combined, the consideration of positive links is relatively simple. This method explores the influence of social attributes and historical usage on workflow fragment recommendation, but ignores the role of social connections in recommendation work, and does not explore the impact of social relations on workflow segment discovery and recommendation.

### 5.3 Syntax-based workflow fragment discovery

The syntax-based method focuses on the structure of workflows and the problem of service composition is regarded as a service matching problem. The matching of interface parameters is adopted as the most important metric to promote the composition. Niu et al. (2016) modeled the workflow fragment discovery problem as an uncertain web service composition planning problem. A total of two new uncertain planning algorithms using heuristic search are proposed, called UCLAO* and BHUC, which use the similarity of service interface parameters to solve the U-WSC planning problem of reduced state space, thereby improving the efficiency of finding service portfolio solutions. Empirical experiments are carried out based on running examples in E-commerce applications and large-scale simulation data sets. However, it does not take the level of expertise of different users into account. In fact, there may exist users who do not know the details of the interface, and may not be able to provide input or output parameters. Moreover, the lack of considering service semantics and social associations may not ensure the correctness of the workflow from a functional point of view.

Due to the fast increase of web services over the Internet, Lin et al. (2012) proposed a backward planning method to discover reasonable workflow fragments in a large-scale web service repository based on the lowest cost. The authors exploit the similarity of input and output parameters to construct service groups for facilitating service search. Also, a backward strategy is used to reduce the search space, in order to improve the computational efficiency during workflow construction. However, this approach also neglects the important functional semantics of services and lacks the exploration of the impact about social association among services on their combination.

Liu et al. (2014) proposed a workflow-based framework for workflow fragments discovery. It not only uses the matching degree of the interface parameters to facilitate service composition but also employs a data-centric composition principle that the parameters matching are based on the tag-based semantics. Also, the semantics of service are determined by the folksonomy. The authors first used the related tags to stand for parameters and then constructed workflows based on them. This approach considers the semantic information of the service as well and can better reflect the functionality of the services. Therefore, it can facilitate the combination of services and the recommendation of workflow fragments from a functional perspective. In fact, besides labels, there is other rich semantic information in the repository that can facilitate the construction of workflow fragments. However, these semantic information are not used. Meanwhile, social repositories contain a rich variety of social information and social correlations among items, and these social correlations are not considered in this approach.

## 6 Conclusion

Considering the knowledge-intensiveness, effort-consuming, and error-proneness when constructing a novel bioinformatic workflow from scratch, discovering and reusing the best practices in legacy workflows is promising when it comes to accomplishing similar tasks. Traditional methods are proposed to discover appropriate workflow fragments depending on their profiles or partial social information in service repositories. However, social relations between developers have not been explored extensively. To capture these relations, this study constructs a knowledge graph *S* ^2^ *KG* that includes two types of entities and three types of relations. Based on *S* ^2^ *KG*, we propose a bioinformatic workflow fragment discovery mechanism, where we identify positive and negative links for service composition through analyzing their co-invocation possibilities and co-developer relations. A SINet is formed by *isInk* relations in *S* ^2^ *KG* to facilitate single service discovery. Finally, the *Yen*’s method is adopted to construct bioinformatic workflow fragments with respect to user’s requirements. Experimental results demonstrate that our method performs better than the state-of-the-art techniques with higher accuracy and efficiency.

## Data Availability

Publicly available data sets were analyzed in this study. This data can be found at: https://www.myexperiment.org/workflows.

## Footnotes

1
https://www.myexperiment.org/workflows
